# Chronic stress and the IL-10-mediated immunoregulatory loop in the pathogenesis of periodontitis

**DOI:** 10.1042/CS20256843

**Published:** 2025-11-25

**Authors:** Maksym Skrypnyk, Axel Spahr, Shlomo Berkovsky, Tetiana Yatsenko, Chun Xu, Olga Zuieva, Taro Osada, Satoshi Takahashi, Nobutaka Hattori, Kazuhisa Takahashi, Koichi Hattori, Beate Heissig

**Affiliations:** 1 Centre for Health Informatics, Australian Institute of Health Innovation, Faculty of Medicine, Health and Human Sciences, Macquarie University, Sydney, NSW, 2109, Australia; 2 Charles Perkins Centre, The University of Sydney, Sydney, NSW, 2050, Australia; 3 Sydney Dental School, Faculty of Medicine and Health, The University of Sydney, Sydney, NSW, 2010, Australia; 4 Juntendo Biomedical Research Core Facilities, Juntendo University Graduate School of Medicine, Tokyo, 113-8421, Japan; 5 Discipline of Periodontics, School of Dentistry, Faculty of Medicine and Health, The University of Sydney, Sydney, NSW, 2010, Australia; 6 Department of Enzymes Chemistry and Biochemistry, Palladin Institute of Biochemistry of NAS of Ukraine, Kyiv, 01054, Ukraine; 7 Department of Gastroenterology, Juntendo University Urayasu Hospital, Urayasu-shi, Chiba, 279-0021, Japan; 8 Division of Clinical Precision Research Platform, The Institute of Medical Science, The University of Tokyo, Tokyo, 108-8639, Japan; 9 Center for Genome and Regenerative Medicine, Juntendo University, Graduate School of Medicine, Tokyo, 113-8421, Japan; 10 Department of Hematology/Oncology, The Institute of Medical Science, The University of Tokyo, Tokyo, 108-8639, Japan

**Keywords:** cytokines, corticotropin-releasing hormone, interleukin-10, hypothalamo-hypophyseal system, periodontitis, stress

## Abstract

Periodontitis is a chronic inflammatory condition that gradually destroys the tissues supporting the teeth, including the gingiva, periodontal ligament, and alveolar bone. Emerging evidence suggests that psychological stress plays a significant role in the initiation and progression of periodontal disease, primarily through its impact on immune regulation. Stressors activate the hypothalamic–pituitary–adrenal (HPA) axis, leading to the release of corticotropin-releasing hormone (CRH) from the hypothalamus and, in turn, adrenocorticotropic hormone (ACTH) from the pituitary gland. Activation of the HPA axis and the sympathetic-adrenal-medullary (SAM) system during stress triggers the systemic release of cortisol, epinephrine, norepinephrine, and cytokines. The HPA, SAM, and cytokines interact in both direct and indirect ways. Not only does stress induce interleukin-10 (IL-10), but IL-10 also helps regulate the stress response and cortisol levels. IL-10 can stimulate the release of CRH and ACTH, while concurrently inhibiting cortisol secretion from the adrenal glands. IL-10 has drawn increasing attention within the oral cavity owing to its dual role in modulating immune responses and maintaining periodontal tissue homeostasis. This review outlines the current understanding of stress-related neuroendocrine pathways and their relevance to periodontal health. It explores the involvement of HPA axis effectors—cortisol and IL-10—in modulating the inflammatory milieu associated with periodontitis. This includes recent insights into IL-10-expressing regulatory B cells and the potential role of IL-10 in mitigating alveolar bone loss. By integrating recent advances in neuroendocrinology, immunology, and oral biology, this review clarifies how systemic stress responses contribute to local inflammatory changes in the periodontium. Understanding the mechanisms linking psychological stress, cortisol dynamics, and IL-10-mediated regulation may offer new opportunities for early diagnosis and intervention in stress-exacerbated periodontitis.

## Introduction

Periodontitis is a long-term inflammatory condition that leads to the gradual destruction of tooth-supporting structures. The destroyed structures include the gingiva, periodontal ligament, cementum, and alveolar bone [1]. Excessive, unregulated, and persistent inflammation leads to irreversible damage of hard and soft tissues, referred to as periodontitis [2]. The prevalence of periodontitis in dentate adults is approximately 62%, and severe periodontitis comprises 23.6% [3].

Gingivitis is a reversible inflammatory condition confined to the gingiva that precedes the development of periodontitis. It arises due to the immune response to the microbial subgingival biofilm. The transition from periodontal health to periodontitis establishes a dysbiotic microbial community that collectively shapes the host immune response, contributing to the progression of periodontitis [4–6]. Various risk factors for periodontitis are outlined in the recent classification of periodontal diseases and conditions, and this includes smoking, diabetes mellitus, socioeconomic factors, obesity, drug use, and stress [2,7–10]. Stress disrupts immune regulation and contributes to poor oral health by altering inflammatory responses, inducing immunosuppression, modifying the bacterial composition of periodontal pockets, and influencing behavioral factors that promote periodontitis [11,12].

**Figure 1:**
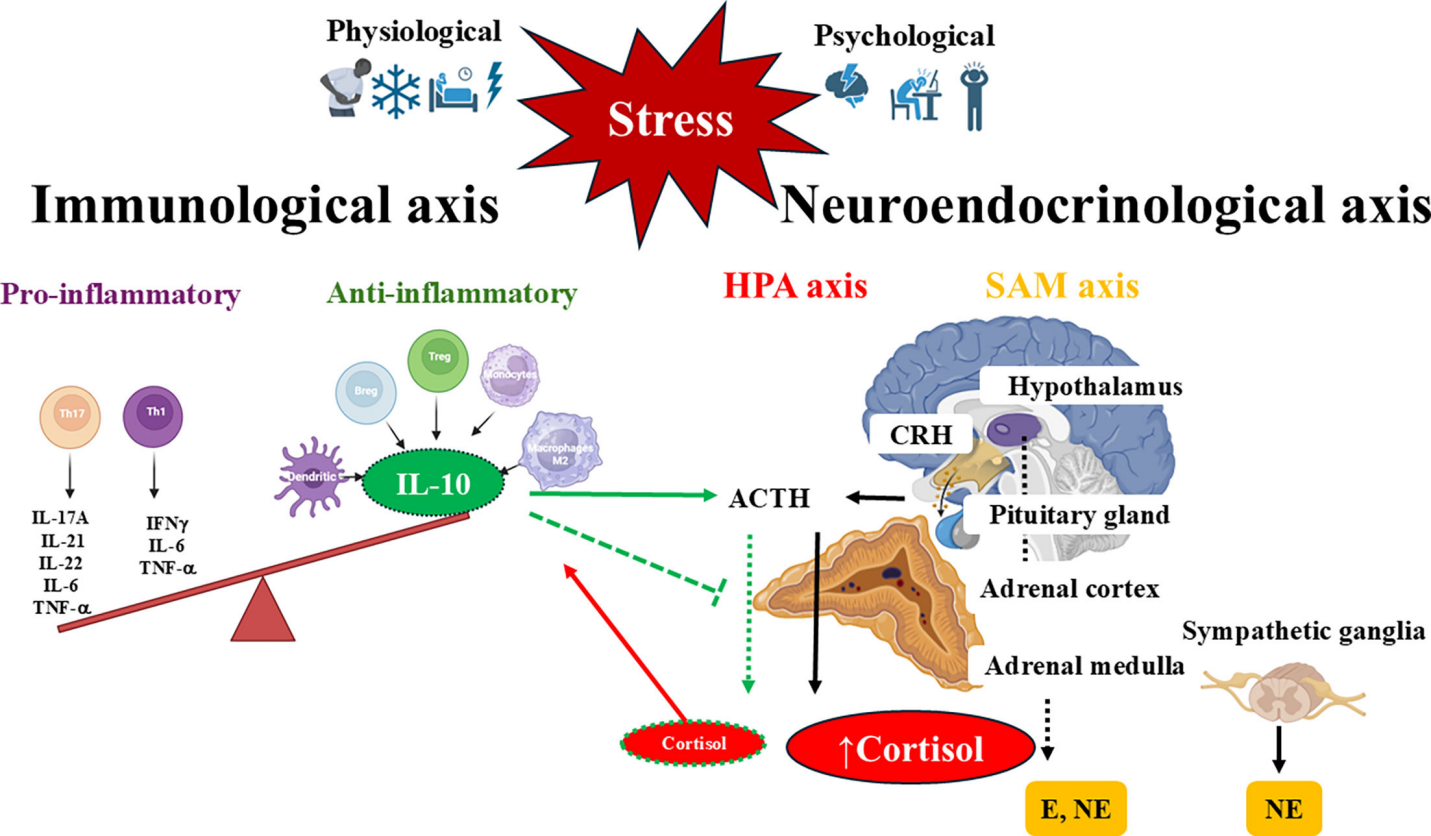
Physiological and psychological stressors activate the hypothalamic–pituitary–adrenal (HPA) axis and the sympathetic–adrenal–medullary (SAM) axis, initiating neurohormonal and immunological responses, with interleukin 10 (IL-10) modulating neuroendocrine activity. Stressors trigger the release of corticotropin-releasing hormone (CRH) from the hypothalamus, which stimulates the anterior pituitary to secrete adrenocorticotropic hormone (ACTH). ACTH then promotes the synthesis of cortisol in the adrenal cortex. The SAM system contributes to the stress response by secreting catecholamines, including epinephrine (**E**) and norepinephrine (NE), from the adrenal medulla and sympathetic ganglia. Chronic stress induces immune dysregulation by enhancing the release of pro-inflammatory cytokines from T helper cells (Th1 and Th17) and suppressing anti-inflammatory cytokines from leukocyte subtypes (dendritic cells, monocytes, M2 macrophages, T and B regulatory cells), which are essential for resolving inflammation. The anti-inflammatory cytokine IL-10 can stimulate CRH production in the hypothalamus and ACTH release. In the presence of ACTH, it suppresses cortisol production in adrenocortical cells, thereby contributing to the maintenance of immune homeostasis under stress conditions.

Research indicates that emotional stress can influence the immune system via the neural and endocrine systems. The neurological stress response involves activating the central nervous system through the hypothalamic–pituitary–adrenal (HPA) axis and/or the sympathetic nervous system (SNS), allowing an organism to adapt physiologically to a threat (Figure 1). Stressors can vary in duration, from acute to chronic, altering the neurological, hormonal, and immune response, all of which alter the clinical onset and progression of periodontitis [7,11,13].

Markers associated with stress and periodontitis are present in both saliva and gingival crevicular fluid (GCF), including cortisol, α-amylase, β-endorphin, chromogranin, salivary IgA, and reactive oxygen metabolites [14–18]. The severity of chronic periodontitis has been shown to correlate with elevated salivary cortisol levels and other effectors of the HPA axis [19–22]. Clinical studies demonstrated that chronic stress can alter the host immune response, increase susceptibility to periodontitis, and promote biofilm—a structured bacterial community on teeth—accumulation [23–25].

Stress up-regulates the effector of the neuronal stress axis cortisol, which has been shown to alter the cytokine balance [26,27]. Stressors like cortisol can increase the production of pro-inflammatory cytokines associated with oral cavity diseases, thereby disrupting immune homeostasis [28–30]. While pro-inflammatory cytokines have been extensively studied in periodontitis, the role of anti-inflammatory mediators such as interleukin-10 (IL-10) and stress hormones like cortisol remains comparatively underexplored, despite emerging evidence of their relevance [31,32].

IL-10 is an anti-inflammatory cytokine that inhibits the production of pro-inflammatory cytokines such as tumor necrosis factor-alpha (TNF-α), interleukin-1 beta (IL-1β), and interleukin-6 (IL-6), which are involved in tissue destruction and disease progression [32,33]. IL-10 and its receptor can be produced in pituitary and hypothalamic tissues [34,35]. IL-10 is released from several cell types, including astrocytes and microglia, after immune challenge and brain injury [36,37]. IL-10 is implicated in oral cavity diseases, including periodontitis and salivary gland disorders [38]. The importance of IL-10 and IL-17 in the pathogenesis of periodontitis has recently been reviewed and will therefore not be covered in this review [39].

This narrative review explores the multifactorial aspects of stress, including changes in the hormone cortisol and the cytokine IL-10. It investigates how these humoral and immune responses contribute to the pathogenesis of periodontitis. We review the pathogenesis of periodontitis, the basis of a neurological stress response leading to cortisol elevation and activation of the inflammatory response that can be blocked by the cytokine IL-10. This review contains studies demonstrating the unique interplay between immunological and neuroendocrinological pathways in the pathogenesis of periodontitis.

### The sympathetic–adreno–medullary system and cortisol

Periodontal disease is more prevalent and severe in individuals extensively exposed to chronic psychological stress [40,41]. In response to a perceived threat, the body’s stress–response systems are activated to maintain homeostasis and ensure survival. The rapid SAM and the slower HPA axis are heavily implicated in the stress response, releasing catecholamines and glucocorticoids accordingly [42]. Activation of the SAM system is clinically manifested by elevated blood pressure, increased heart rate, and altered rheological parameters. This response can be influenced by systemic factors such as obesity and is associated with periodontal disease [43]. There are gender differences in the HPA axis and SAM reactivity, with males exhibiting a greater stress response [44]. A functional neuroimaging study has demonstrated that emotional stress triggers the activation of distinct neural networks in both male and female individuals [45]. These sex-specific neural responses may facilitate optimal emotional stress regulation. However, in rodents, basal cortisol levels are higher in females, whereas in humans, they are higher in males. Despite this, women exhibit greater sensitivity to cortisol, whereas no gender differences are observed in pituitary function [44,46–48].

The hypothalamus is the brain’s control center for the stress response, which activates the HPA axis and the SAM system (Figure 1). The SAM response to stress is manifested through the production of the catecholamines epinephrine (E) and norepinephrine (NE) by the adrenal medulla and sympathetic ganglia [30,49]. E and NE are acute stress markers of the activated SAM system [50].

Concurrently or shortly following the SAM response, the paraventricular nucleus of the hypothalamus, the primary center for processing stressors, secretes CRH into the bloodstream. The CRH hormone acts on target organs containing CRH receptor-1 (CRH-R1), which is primarily expressed in brain structures such as the anterior pituitary gland, cerebral cortex, basal ganglia, hippocampus, and cerebellum, as well as in endocrine glands including the thyroid, adrenal glands, pancreas, and testes. Additionally, CRH receptor-2 is predominantly found in the brain (choroid plexus, cerebral cortex, and hypothalamus) and in endocrine organs (pituitary and thyroid, the heart, skeletal muscles, and tongue) [51]. When stimulated by CRH, the anterior part of the pituitary gland releases ACTH, which regulates cortisol production in the zona fasciculata of the adrenal cortex (Figure 1). Cortisol exerts a negative feedback effect by binding to glucocorticoid receptors (GR) in the hypothalamus and pituitary, thereby inhibiting further production of CRH and ACTH.

The HPA and SAM systems work together to regulate acute stress. Cortisol (from the HPA axis) can enhance sensitivity to catecholamines like epinephrine [52]. At high levels, cortisol can inhibit further sympathetic activity, providing negative feedback to prevent overactivation. Similarly, catecholamines (from the SAM axis) can stimulate CRH release, potentially boosting HPA activity early in the stress response [53].

Connecting the neuronal cortisol stress pathway to the cytokine response, cortisol, through glucocorticoid receptor-α (GR-α), can promote the expression of the cytokine IL-10 in monocytes and macrophages. This process helps suppress pro-inflammatory cytokines such as TNF-α, IL-6, and IL-1β, aiding in the resolution of inflammation [54]. Low epinephrine excretors showed two- to five-fold higher LPS-induced TNF-α and IL-12 and two-fold lower IL-10 levels *ex vivo* [55]. IL-10-deficient mice have been shown to develop spontaneous chronic inflammation and autoimmune disorders [56,57] and exhibit elevated basal corticosterone levels compared with their wildtype counterparts [58]. IL-10 affects the adrenal function. In Y-1 adrenocortical cells, IL-10 inhibits the production of glucocorticoids [58].

### Pathogenesis of periodontitis

The transition from periodontal health to periodontitis is multifactorial, involving both a shift in the subgingival microbiota from a symbiotic to a pathogenic state and environmental influences that trigger an altered host immune response [5,59,60]. These environmental challenges include mechanical forces like tooth brushing and mastication, chemical insults like tobacco smoke, and constant interaction with a diverse microbial community. The prevalence and severity of periodontitis are typically higher in males compared with females [61,62]. Periodontitis is typically characterized as a chronic inflammatory disease that damages the supporting structures of the teeth within the alveolar bone. It is driven by reciprocally reinforced interactions between the dysbiotic microbiome and dysregulated inflammation. These tissues include the gums, periodontal ligament, root cementum, and alveolar bone. The development of dysbiotic bacterial communities, along with the host’s inflammatory response, creates a self-sustaining feed-forward loop that perpetuates periodontitis.

Microbial factors play a central role in the pathogenesis of periodontitis. Bacteria like *Porphyromonas gingivalis* (*P. gingivalis*) act as a keystone pathogen of periodontitis, which, collectively with other ‘accessory pathogens’ such as *Streptococcus gordonii, Tannerella forsythia,* and *Aggregatibacter actinomycetemcomitans*, disrupt the homeostasis of the subgingival microbiota [4]. A dysregulated immune response enables periopathogens to thrive in the oral cavity. *P. gingivalis* can evade the immune response by inducing TLR2-dependent IL-10 production, which further inhibits interferon-gamma (IFN-γ) production by CD4+and CD8 + T cells, resulting in immunosuppression [63]. The status of the host immune response is a critical determinant of the extent to which oral bacteria contribute to the destruction of the periodontal complex.

Chronic periodontitis is clinically characterized by progressive loss of the gingival tissue attachment to the tooth, with the formation of periodontal pockets, degradation of the periodontal ligament, and loss of the alveolar bone. As a result, the progression of this disease can lead to deeper periodontal pockets, gum recession (which exposes the cervical area of the teeth), tooth mobility, occlusal dysfunction, and, ultimately, tooth loss [2]. Clinical symptoms indicating periodontitis include gingival bleeding, pain, discharge from the pockets, alterations in gum shape or consistency, tooth sensitivity and mobility, bad breath, and loss of bone and connective tissue attachment.

The primary goal of periodontitis therapy is to eliminate the etiological factor, bacterial plaque, by mechanically removing it and restoring a balanced oral microbiota through effective biofilm control [64,65]. Adjunctive antibiotic therapy combined with mechanical debridement (scaling and root planing) has demonstrated very low-certainty evidence of efficacy [66]. Moreover, its use is associated with the growing concern of antibiotic resistance in the management of periodontitis [67–69]. Novel strategies targeting signaling molecules, such as the cytokines interleukin-17 (IL-17) and IL-10, have emerged as potential therapeutic options [64,70,71]. The importance of IL-10 and -17 in the pathogenesis of periodontitis has recently been reviewed and will therefore not be covered in this review [39].

IL-10 has emerged as a promising therapeutic molecule for periodontitis due to its ability to down-regulate chronic inflammation-associated cytokines, such as IL-17.

### Neuronal stress molecules as biomarkers for periodontitis

Chronic stress can contribute to periodontitis indirectly through the adrenergic axis (the HPA axis), the behavioral axis (including poor oral hygiene, inadequate nutrition, smoking, changes in eating behavior, and dental parafunction), and the immune response (characterized by poor vascularization, activation of osteoclastogenesis, and secretion of pro-inflammatory factors) [11,72]. The genetics of stress response and stress-related disorders include variations in genes involved in the sympathetic nervous system or the hypothalamic-pituitary-adrenocortical axis that are linked to altered stress responses [73].

Stressed animals with periodontitis experienced higher gingival atrophy [74] and worse clinical symptoms such as gingival bleeding, periodontal pocket depth, plaque index, and tooth mobility [75]. Stress- and inflammation-induced down-regulation of GR-α in periodontal tissues and the hypothalamus of rats dysregulates the HPA axis by elevating systemic cortisol levels that are not effectively mitigated by antidepressant treatment [75,76]. The HPA axis can also directly increase cortisol and/or NE in GCF, promoting periopathogen growth and virulence [77,78].

Cortisol levels may remain chronically elevated in individuals who fail to cope with stress, leading to down-regulation of cellular immune responses [19]. High salivary cortisol levels, following free blood cortisol, are found in patients with chronic stress, even after awakening [79–83]. This straightforward relationship connecting stress, blood, and salivary cortisol makes the latter the biomarker of choice in stress investigation for the non-invasive assessment of free cortisol levels [14].

**Figure 2:**
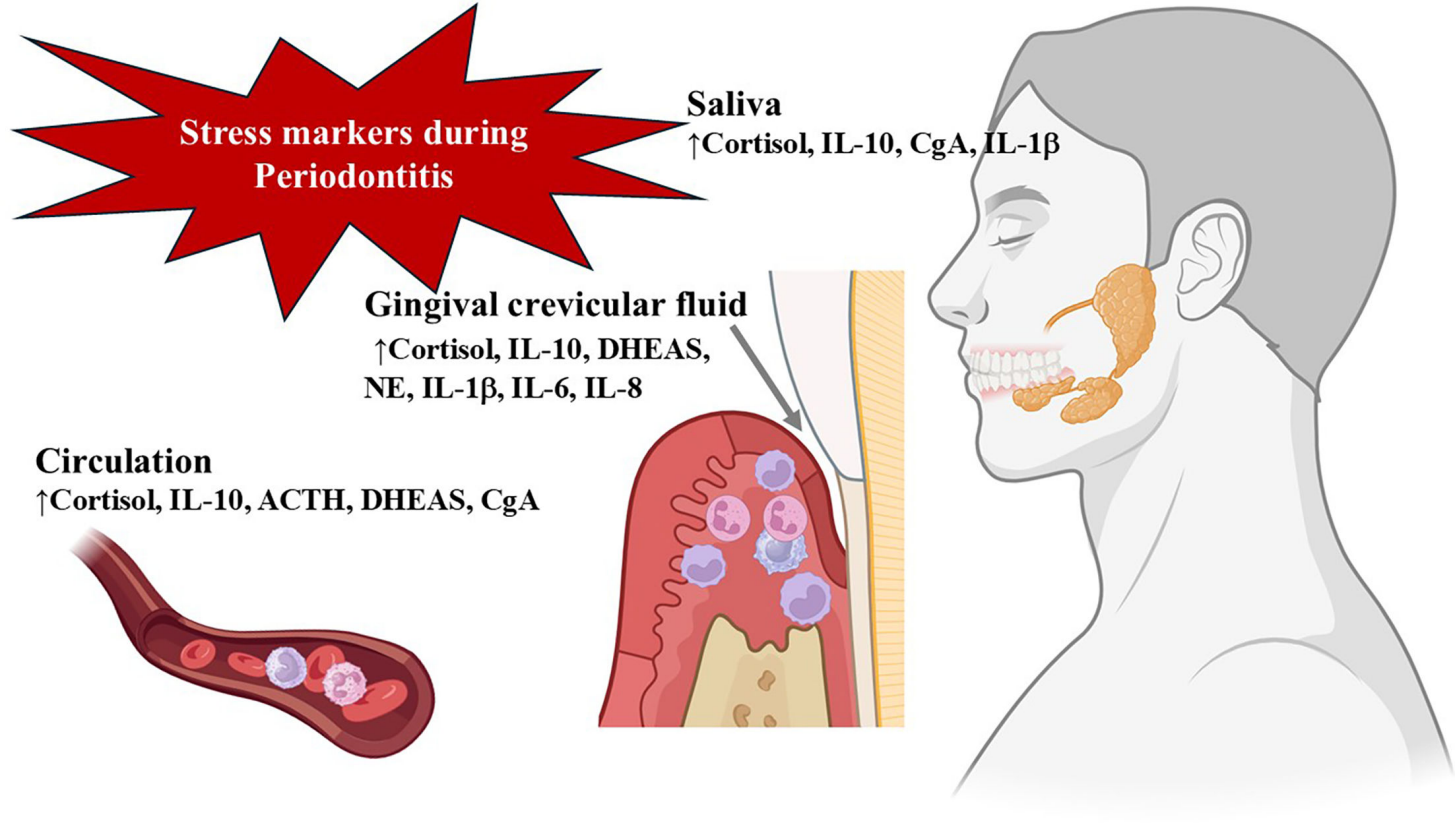
Markers of chronic stress are detectable in saliva, gingival crevicular fluid (GCF), and blood during the progression of periodontitis. Chronic stress activates the hypothalamic–pituitary–adrenal (HPA) axis, releasing cortisol, adrenocorticotropic hormone (ACTH), dehydroepiandrosterone sulfate (DHEAS), and chromogranin A (CgA) into the peripheral circulation. These biomarkers are subsequently detectable in saliva and GCF, reflecting the degree of HPA axis activation. In parallel, norepinephrine (NE) levels, a key product of the sympathetic-adrenomedullary (SAM) system, are also elevated. Additionally, IL-10 levels increase in all these biological fluids in response to chronic stress and periodontal inflammation, indicating its dual role in immunoregulation and stress response.

Cortisol, ACTH, dehydroepiandrosterone sulfate (DHEAS), and CgA, as key markers of the HPA axis, can be detected in saliva, GCF, or peripheral blood (Figure 2).

Increased cortisol levels over time lead to changes in periodontal tissue resistance, thereby raising susceptibility to periodontitis development [41]. Adult patients with aggressive periodontitis showed higher total salivary and GCF cortisol levels [15] and DHEAS in GCF [17]. In patients with aggressive periodontitis, a peak in serum cortisol was observed before nighttime, when cortisol levels are usually lowest [15]. High salivary cortisol was associated with chronic periodontitis in Iranian, Turkish, Peruvian, Colombian, and Indian adults, without any gender difference [21,22,84–86]. High serum cortisol and DHEAS correlated with periodontitis severity in old Japanese adults [87]. No gender differences have been reported in salivary cortisol levels among healthy individuals; however, younger individuals exhibit greater HPA axis activation in response to psychosocial stress [88].

Inconclusive results were obtained regarding the diagnostic value of GCF cortisol. In Turkish adults, no correlation was found between GCF cortisol levels and anxiety/stress or periodontitis; however, the level of DHEAS in GCF correlated with the severity of periodontitis [16]. Using data from 3388 participants of two population cohort studies within the Study of Health in Pomerania project, cortisol levels were positively associated with follow-up mean clinical attachment level (CAL), deep interdental CAL, and bleeding on probing. Still, they were unrelated to the mean probing pocket depth and the presence of deep periodontal pockets. This prospective observational study did not find robust evidence for the effect of cortisol on periodontitis [89]. Similarly, Schmidt et al. found no relationship between blood cortisol, DHEAS, and periodontitis among German adolescents; however, they suggested a possible association between DHEAS and matrix metallopeptidase-8, which may be linked to the severity of inflammation [90].

Under the influence of cortisol, fibroblasts exhibit increased fibronectin deposition, which facilitates the adhesion of *P. gingivalis* and other oral bacteria [91]. There is also a concomitant reduction in T-cell infiltration, potentially creating a microenvironment that supports bacterial colonization and impairs local immune surveillance [92,93].

### Stress-related regulation of IL-10 and IL-10’s feedback on the HPA axis

During stress, the central nervous, endocrine, and immune systems interact in a complex and co-ordinated manner [13,29]. There may be a potential interaction between chronic stress and the anti-inflammatory cytokine IL-10 [94]. Psychological stress increases IL-10 and cortisol levels, and a similar effect is observed in glucocorticoid-treated neutrophils [25,93]. This suggests that IL-10 might be a key mediator of stress-induced immunosuppression [95].

The sympathetic neurotransmitter NE increased LPS-induced IL-10 and IL-19 expression in splenocytes and dendritic cells, indicating its role as a regulator of a stress-induced immune response. NE’s ability to induce these cytokines’ expression was blocked by pre-treatment with the beta-adrenoceptor antagonist propranolol [96]. In another study, pre-treatment of mice with beta-adrenoreceptor agonist (nadolol) or benzodiazepine anxiolytic (chlordiazepoxide) prevented the stress-induced IL-10 expression [95,96].

Not only does stress induce IL-10, but IL-10 also regulates the stress response and glucocorticoid levels. In the hypothalamic-pituitary cytokine network, IL-10 can inhibit the expression of pro-inflammatory cytokines IL-1β, TNF-α, and IL-6 [97], which in turn stimulate the release of ACTH and CRH. This underscores the bidirectional communication between the immune and neuroendocrine systems [98].

IL-10 has a complex and context-dependent interaction with ACTH, acting as both a stimulator and inhibitor of the HPA axis. In some contexts, IL-10 has been shown to stimulate CRH production in the hypothalamus and ACTH release from the pituitary gland, while concurrently inhibiting glucocorticoid (cortisol) secretion from the adrenal glands [99]. IL-10 acts on cells of the zona fasciculata in the adrenal glands, where IL-10 receptor alpha (IL-10Rα) is highly expressed [58].

Conversely, IL-10 in the presence of ACTH, but not on its own, decreases glucocorticoid production in the adrenal gland [58]. Glucocorticoids are produced from cholesterol through a carefully regulated process involving key enzymes involved in steroidogenesis. Cholesterol is initially transported into the mitochondria by the steroidogenic acute regulatory protein (StAR), where it is converted to pregnenolone by the rate-limiting enzyme P450scc. Pregnenolone is then transformed into progesterone by 3β-hydroxysteroid dehydrogenase (3β-HSD), marking the start of downstream steroid biosynthesis [100]. It has been shown that IL-10 in the adrenal gland reduces glucocorticoid production by down-regulating steroidogenic enzymes, including P450scc and 3β-HSD, as well as the StAR [58]. In the presence of ACTH, IL-10 acts as an essential feedback mechanism within the HPA axis, helping to regulate immune responses and neuroendocrine function.

Glucocorticoids, in turn, increase IL-10 production, which helps suppress the HPA axis by reducing the body’s stress response. This is part of a negative feedback loop where rising glucocorticoid levels from an initial stressor activate the anti-inflammatory cytokine IL-10, which helps to ‘turn off’ the stress response.

Sex differences have been observed in regulators of the HPA axis (glucocorticoids) and cytokines, such as IL-10; however, this review cannot cover this subject. A recent study showed that IL-10-producing monocytes play a role in sex differences in pain resolution in both mice and humans [101]. Likewise, the HPA axis displays notable sex-biased activity [102].

 Administration of exogenous IL-10 reversed stress-induced behavioral changes, underscoring its neuroprotective role [103]. IL-10 is enhanced at transcriptional and protein levels in stressed mice [104]. IL-10 levels increase for up to 24 h following acute stress in study subjects induced by physical exercise, preceding a rise in cortisol levels [105].

In the absence of functional IL-10—IL-10 deficiency—regulation of IL-10, glucocorticoids, and the HPA axis are thrown into disarray. IL-10-deficient mice have higher serum corticosterone than wildtype mice under basal conditions, but no changes in ACTH levels [99].

The cytokine milieu affects B cell phenotype. High levels of pro-inflammatory cytokines, particularly IL-6 and low concentrations, are associated with reduced CD5 expression and impaired differentiation of B cells into antibody-producing plasma cells, which may exacerbate periodontal tissue damage [106–108].

Higher IL-10 levels indicate an anti-inflammatory response [109]. Elevated levels of IL-10 and cortisol in GCF and saliva are associated with severe periodontitis [15,17,110]. Salivary IL-10 levels were lower in patients with unstable periodontitis. In contrast, those with stable periodontitis and the control group had similar IL-10 levels, suggesting that the exacerbation of periodontitis is associated with reduced IL-10 levels [111]. The levels of IL-10 and IL-18 were significantly higher in the advanced periodontitis group than in the healthy or mild periodontitis groups [109,112]. Surprisingly, higher baseline IL-10 levels in GCF have been linked to improved clinical outcomes following treatment in patients with aggressive periodontitis [113].

High-intensity training has demonstrated protective effects against stress-aggravated periodontitis in animal models by reducing alveolar bone loss, preserving bone mineral density and microarchitecture, lowering serum TNF-α, and enhancing IL-10 levels compared with untrained animals subjected to stress [114].

In the following section, we will discuss recent developments on the role of IL-10 and IL-10-expressing regulatory B cells (Breg) in the pathogenesis of periodontitis.

### IL-10 signaling and receptor interaction and its modulation of NF-kB signaling

IL-10, a potent anti-inflammatory cytokine, is secreted by various immune cells, including macrophages, dendritic cells, T regulatory cells (Tregs), and B cells [115]. IL-10 mitigates excessive inflammation and tissue damage, serving as a protective mechanism that preserves host tissue integrity [116]. IL-10 exerts its effects by binding to the IL-10 receptor (IL-10R), a heterodimer composed of two subunits. The IL-10Rα is mainly expressed in immune cells, while the IL-10Rβ is ubiquitously expressed and shares homology with other cytokine receptors, such as IL-22 and IL-26 [97]. When IL-10 binds to its receptor, the IL-10R complex activates the Janus kinase/signal transducer and activator of transcription (JAK/STAT) pathway, particularly STAT3 [117,118]. Poholek et al. reported that IL-10 induced the transcription factor Blimp-1 in Th2 cells through STAT3. STAT3 and Blimp-1 then acted together to boost IL-10 expression in a positive regulatory loop [119]. A gender difference in IL-10 signaling has been observed in male leukocytes, with increased STAT3 activation leading to reduced TNF-α production after LPS stimulation [120]. Inhibition of macrophage proliferation and monokine production by IL-10 is governed by two distinct signaling pathways, the former requiring STAT3 [121]. IL-10 can be induced in response to Toll-like receptor 2 and 4 (TLR2 and TLR4) activation, described mainly in cells of the innate immune response; dendritic cells and macrophages [122,123].

MicroRNA-466l has been implicated in regulating IL-10 expression. Its expression and IL-10 mRNA expression are reduced in periodontal ligament stem cells derived from periodontitis-affected teeth. Transfection with microRNA-466l enhanced IL-10 levels in healthy and periodontitis-isolated periodontal ligament stem cells and inhibited cell proliferation, regardless of the periodontal ligament stem cells’ origin [124].

Optimal TLR-induced IL-10 production requires both cytosolic TLR adaptor proteins, myeloid differentiation primary response gene (MyD88), and TIR-domain-containing adapter-inducing interferon-β, indicating a role for Type I interferons, NF-κB, and mitogen-associated protein kinase in IL-10 transcription [125].

A close interaction among STAT3, IL-10, and NF-κB signaling has also been observed [126,127]. The NF-κB signaling pathway comprises a canonical and a noncanonical pathway. Canonical NF-κB is rapidly activated, leading to the production of proinflammatory mediators that promote inflammation, immune cell activation, differentiation, and apoptosis. However, its abnormal activation can contribute to chronic inflammation, cancer development, and autoimmune diseases [128]. NF-κB activation can be triggered by various stimuli, including TNF-α, IL-1β, bacterial LPS, pathogen-associated molecular patterns via TLRs, B- and T-cell receptors, CD40L, and RANKL [128,129]. These stimuli activate IκB kinase complex, leading to the phosphorylation and breakdown of IκBα, the inhibitor of NF-κB. This enables the p65 (RelA)/p50 dimer to enter the nucleus and initiate transcription of target genes involved in inflammation, immunity, and cell survival [128]. The activation of the non-canonical NF-κB pathway occurs through a few members of the TNF receptor superfamily [128].

IL-10 blocks NF-kB activity at two levels: through the suppression of IκB kinase activity and through the inhibition of NF-kB DNA binding activity [130]. Pretreatment with IL-10 blocks TNF-α–induced nuclear translocation of p65 but not p50. Experiments in *p105/p50*-deficient mice showed that IL-10 inhibits cytokine production (MIP-2α, IL-6) only in wildtype macrophages, indicating that IL-10’s anti-inflammatory action depends on activation of the repressive p50/p50 NF-κB homodimer [131].

IL-10-deficient mice have altered A20 and IκBα synthesis, which are key negative regulators of the NF-κB pathway [132]. In monocytes stimulated with LPS or TNF-α, IL-10 inhibits NF-κB activation by blocking its nuclear translocation, thereby suppressing the expression of inflammatory cytokine genes [127]. Pharmacological inhibitors of NF-κB, such as tosylphenylalanyl chloromethyl ketone and pyrrolidinedithiocarbamate, cause similar suppression of cytokine transcription in LPS-stimulated monocytes [127].

The NF-κB transcription factor plays a crucial role in the development of periodontitis by mediating the inflammatory response [133]. Periodontal pathogens such as *Fusobacterium nucleatum* and *P. gingivalis* activate NF-κB signaling through components like p65, phospho-p65, the IκB kinase complex, and the phospho-IκB kinase complex [134,135]. Experimental studies have confirmed this activation, showing increased production of inflammatory mediators, up-regulation of NF-κB transcription factors (p50/p65), and inhibition of IκB expression in the gingival tissues of patients with periodontitis [136]. Increased phosphorylation of p65 in the NF-κB signaling pathway within periodontal ligament cells boosts the production of TNF-α, IL-1β, and IL-6 [137]. Blocking NF-κB signaling in periodontal tissues, periodontal ligament cell lines, or alveolar bone reduces pro-inflammatory cytokines (IL-1β, IL-6, and TNF-α), helps maintain bone levels, and supports normal bone formation [138–141]. miR-21 aggravates periodontitis by increasing the expression of phosphorylated NF-κB and reducing total IκB levels, both in vivo and in vitro [142]. Impaired bone formation was observed in mice with a double knockout of the *NF-κB1* and *NF-κB2* genes, which led to the development of osteopetrosis [143,144].

Overall, these studies show that a decrease in IL-10, as can occur during stress, activates the proinflammatory NF-κB pathways.

### IL-10-secreting B (B10 or breg) cells in periodontitis

Following infections with pathogens, their clearance is controlled by the inflammatory response [145]. In chronic inflammation, such as periodontitis, the immune system is constantly activated. A population of suppressor B cells (Bregs) has been associated with excessive inflammation. Inflammation is self-limiting and regulated by the release of cytokines, such as IL-10, which aid in resolving the inflammatory response [146]. Periodontitis is a chronic inflammatory condition accompanied by a defective immune response. Clinical samples from patients with periodontitis showed elevated levels of immunoreactive B10 cells and IL-10 expression in inflamed gingival tissues [147]. Experimental periodontitis models have confirmed a significant increase in CD45^+^IL-10^+^ cells within diseased gingiva compared with healthy controls [148].

Bregs are distinct subpopulations of B cells that possess immune-regulating and immune-suppressing capabilities, thereby contributing to peripheral tolerance. They can inhibit pro-inflammatory cytokine responses while facilitating the differentiation of Tregs [149]. Most IL-10-producing Bregs, initially characterized as CD19^+^CD1dhi CD5^+^IL-10, are referred to as B10 cells because they are the predominant producers of IL-10 among B cell subsets [150]. These cells have shown significant anti-inflammatory effects in collagen-induced arthritis and colitis [151,152].

Bregs can be activated by TLR2, TLR4, TLR9 agonists, heat shock proteins, and CD40L [153,154]. Stimulation with *P. gingivalis* LPS (a TLR4 agonist) and CpG increases the percentages of IL-10-expressing CD1dhi CD5^+^ B cells in *P. gingivalis*-associated ligature-induced experimental periodontitis [155]. It was demonstrated that the adoptive transfer of CD1dhi CD5+B cells inhibited periodontal bone loss in *P. gingivalis*-associated ligature-induced experimental periodontitis, suggesting a regulatory role of B10 cells in periodontitis and a potential novel principle for treating periodontal diseases. The adoptive transfer of this B cell subpopulation suppressed gingival expressions of RANKL/OPG, TNF-α, and IL-1β, while promoting gingival IL-10 production [155]. In human studies, CD19^+^CD24^+^CD38^+^ Bregs were more prevalent in the peripheral blood of patients with stage II periodontitis and were positively correlated with elevated levels of IL-10, TGF-β, and IL-35 [156].

### IL-10 polymorphism and periodontitis

The IL-10 gene spans about 4.7 kb on chromosome 1q21–32 and contains five exons separated by four introns [157]. Genetic regulatory mechanisms tightly control the expression of IL-10. Several single-nucleotide polymorphisms (SNPs), including -1082A/G (rs1800896) and −592 A/C (rs1800872), are localized within the promoter region of the IL-10 gene that influence IL-10 expression levels.

**Figure 3:**
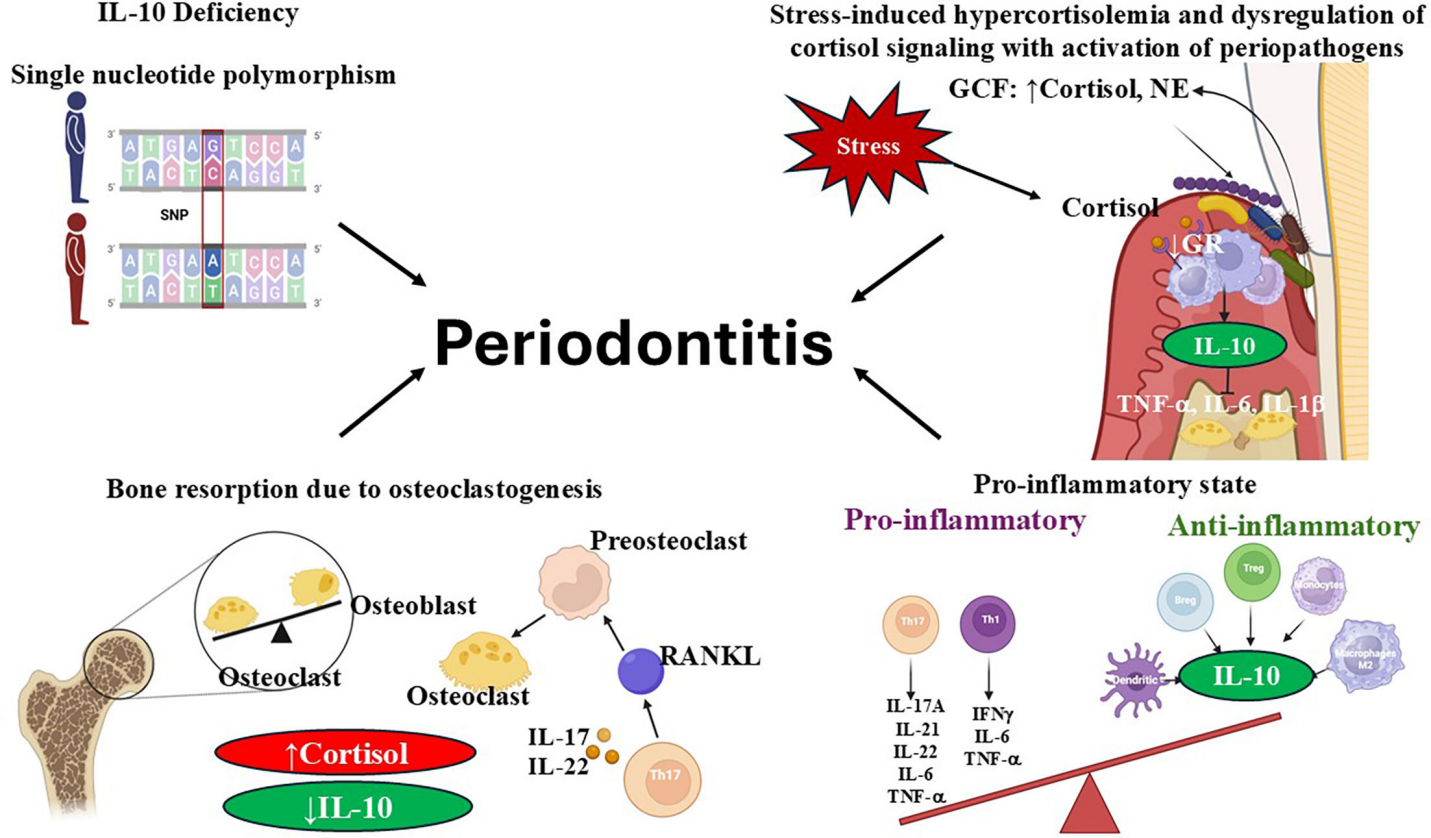
Stress–driven down-regulation of IL-10 contributes to periodontitis. IL-10 deficiency, often associated with single-nucleotide polymorphisms (SNPs) in the IL-10 gene, influences basal cortisol levels, promoting osteoclastogenesis and subsequent alveolar bone resorption. Stress contributes to the development of a chronic pro-inflammatory state. Chronic hypercortisolemia and bacterial inflammation disrupt cortisol signaling in periodontal tissues by down-regulating glucocorticoid receptor (GR) expression, which is essential for IL–10–mediated anti-inflammatory responses. Elevated cortisol and norepinephrine (NE) levels in gingival crevicular fluid (GCF) have been shown to enhance the proliferation and pathogenicity of periodontopathogens, further exacerbating periodontal disease progression. IL-10 deficiency results in the activation of Receptor activator of nuclear factor kappa-Β ligand (RANKL)- secreting T-helper 17 (Th17) cells, which promote osteoclast differentiation. Osteoclasts play a crucial role in bone resorption and loss in periodontitis.

Studies have shown that individuals with low IL-10-producing genotypes, specifically those with IL-10 polymorphisms, are more susceptible to severe periodontitis due to a heightened inflammatory response [158]. The -819C > T(rs1800871) and -592C > A(rs1800872) polymorphisms were associated with an increased risk of chronic periodontitis in Latinos [159] (Figure 3). In the Iranian population, IL-10–592 C/A, IL-10–819 C/T genes were significantly associated with periodontitis [160]. Similar findings were reported in the Chinese Han population, where individuals with the IL-10–592 AA and -819 TT genotypes showed higher frequencies of periodontitis compared with healthy controls after adjusting for age, gender, and periodontal status [161]. In Asians, no associations were observed for any IL-10 polymorphisms under all comparison models [162]. The −1082A > G(rs1800896) polymorphism affects IL-10 transcriptional activity, with the G allele linked to higher IL-10 production and associated with a decreased risk of chronic periodontitis in Caucasians and Latinos [162,163]. In human gingival fibroblasts, the IL-10 promoter SNP rs6667202 with the CC genotype was associated with higher IL-10 production following bacterial stimulation (compared with the AA and AC genotypes) in individuals with a healthy periodontium. However, in patients with grade C, stage III or IV periodontitis, the same CC genotype had reduced IL-10 expression, suggesting that additional factors may modulate the IL-10 response [164].

### Interaction between IL-10 and IL-17 signaling in periodontitis

IL-17 is a crucial cytokine in the periodontium. Under normal conditions, IL-17 is produced by Th17 cells, innate lymphoid cells, γδ T cells, and natural killer T cells, depending on the inflammatory context, which can range from bacterial to fungal or viral infections. Its anti-inflammatory properties support mucosal immunity functions in host defense by recruiting neutrophils and enhancing phagocytosis. However, excessive IL-17 production in disease states can lead to chronic inflammation, ongoing tissue destruction, and bone loss [70,165]. Overexpression of the Th17/Treg axis plays a critical role in the initiation and progression of periodontitis [166,167]. In periodontitis, Th17 cells exacerbate disease pathology by driving the production of pro-inflammatory cytokines and tissue destruction [168]. Recent experimental findings indicate that IL-17 stimulates osteoclast activity in the early phases of experimental periodontitis, while potentially providing bone protection in later stages [169].

IL-10 dampens excessive IL-17 signaling and fibrinolysis (breakdown of fibrin) and prevents inflammatory bone destruction [165,170]. It was shown that IL-10 signaling in regulatory T cells is required to suppress Th17 cell-mediated inflammation [171] (Figure 3). IL-10 transcript levels were elevated in the gingiva of patients with periodontitis, and the IL-17/IL-10 ratio was increased in their gingival tissues [158]. It was shown that IL-10-deficient mice presented superior bone loss, accompanied by higher *IL-17* and IL-17–mediated chemokine and cytokine transcript levels compared with control mice after induction of experimental ligature-induced periodontitis with *P. gingivalis* [158]. More IL-17-secreting T cells (CD45 + CD3 + RORγt + IL-17+) were found in the lymph nodes and spleens of IL-10^-/-^ mice than in WT mice, indicating that baseline IL-17 activity is elevated in IL-10^-/-^ mice. Gingival tissues of patients with periodontitis exhibited high levels of IL-10 and RANK transcripts, as well as an increased IL-17/IL-10 ratio [158]. Injection of neutralizing IL-17 antibodies in IL-10^-/-^ mice reduced alveolar bone loss (ABL) due to experimental periodontitis [158]. It was proposed to use IL-10 and IL-10 Rα to attenuate excessive IL-17 activity, thereby reducing the clinical expression of periodontitis and ABL and maintaining homeostasis [158].

### IL-10 reduces bone loss during periodontitis

ABL is a hallmark of periodontitis progression, and preventing it poses a significant clinical challenge in treating periodontal disease [172]. Chronic physiological and psychological stress exacerbates ABL associated with periodontitis [74,96,173–176], in part through the up-regulation of receptor activator of nuclear factor-κB ligand (RANKL) expression in inflammatory cells within the periodontal tissues [177], which leads to osteoclastic resorption [75,174].

Bone remodeling, which involves the breakdown and buildup of bone tissue, is driven by co-ordinated activity of osteoblasts, which build new bone, and osteoclasts, which break down old or damaged bone. RANKL, its receptor RANK, and the decoy receptor osteoprotegerin (OPG) are key molecules regulating osteoclast differentiation, recruitment, and function [178,179] and ABL. RANKL is essential for differentiating osteoclast precursor cells and is critical in periodontal bone resorption [180]. Osteoclasts and RANKL induce periodontal bone resorption. B and T cells are the primary cellular sources of RANKL in the bone resorptive lesions associated with periodontal disease or remodeling [181,182] (Figure 3).

The pro-inflammatory cytokines (IL-1, IL-6, and TNF-α) have been implicated in stimulating osteoclastic resorption in periodontitis. In contrast, IL-10 influences bone loss diseases by inhibiting osteoclast formation [183]. IL-10 inhibited calcium signaling downstream of RANK by inhibiting the transcription of triggering receptor expressed on myeloid cells-2 [184].

Research shows that IL-10 selectively inhibits osteoclastogenesis by preventing the differentiation of osteoclast progenitors into preosteoclast-like cells in rat bone marrow culture systems [185]. Additionally, it promotes osteoblastic differentiation by reducing transforming growth factor-beta 1 production [186]. Animal studies confirm that a lack of IL-10 leads to femur bone loss [187] and ABL [188,189], providing further evidence of IL-10’s role in bone metabolism.

IL-10 up-regulates concentration-dependent OPG expression in human periodontal ligament fibroblasts [190]. Conversely, RANKL expression was reduced in a concentration-dependent manner in IL-10-treated human periodontal ligament fibroblasts. These data suggest that IL-10 is involved in bone remodeling pathways.

IL-10 decreases osteoclastogenesis and ABL in a ligature-induced murine model by down-regulating RANKL [158]. Studies indicate that IL-10 administration impaired ABL in Actinomyces-infected HuPBL-NOD/SCID mice. This impairment was due to decreased RANKL + Th1-associated ABL and the coexpression of human gamma interferon (hIFN-γ) and human macrophage colony-stimulating factor, but not hIL-4, in RANKL + Th cells. This humanized murine model reflects immunological cellular changes in successfully treated patients with underlying aggressive periodontitis [191].

Stress can disrupt the circadian clock, which regulates gene expression, behavior, and physiological functions that depend on daily environmental changes. Studies have shown that disrupting the circadian rhythm in mice impairs the number of bone marrow Treg cells and decreases serum IL-10. It has been demonstrated that administering IL-10 prevents RANKL-induced osteoclastogenesis [192,193]. Melatonin may limit the oxidative and inflammatory effects associated with chronic stress by modulating neurohumoral systems, such as the HPA and SAM [194]. A recent study showed that correcting the circadian rhythm using melatonin protects against periodontitis-associated ABL in stressed rats. This protective effect is achieved through IL-10 up-regulation, RANKL/OPG ratio normalization, and reduced IL-1β levels within the gingival tissues [173].

### Concluding remarks and future research

There is growing recognition that psychological stress contributes to the onset and progression of periodontitis. This review outlined how stress-responsive neuroendocrine pathways, specifically the HPA axis and the SAM system, contribute to immune modulation within the periodontium. Cortisol, a key output of these systems, alters the local immune environment, while IL-10 emerges as a cytokine that dampens pro-inflammatory signaling and preserves tissue integrity. IL-10’s regulatory functions extend beyond immune suppression. The ability of IL-10 to modulate key pro-inflammatory cytokines such as IL-1β, TNF-α, and IL-6, along with its interplay with IL-17 signaling and its regulatory effects on osteoclast differentiation and ABL, points to its multifaceted role in the pathophysiology of periodontitis. The identification of IL-10-producing B cells and evidence for genetic polymorphisms affecting IL-10 expression add further layers to its immunoregulatory significance in periodontal disease.

While salivary and GCF levels of IL-10 and cortisol have emerged as potential non-invasive biomarkers, their clinical application remains uncertain. Inconsistencies in study design, sample collection techniques, and patient heterogeneity have hindered reproducibility and limited their diagnostic value. Future investigations should focus on standardizing analytical protocols and establishing correlations between these biomarkers and disease progression or therapeutic outcomes to advance their use in clinical settings. Its ability to down-regulate cytokines such as IL-1β, TNF-α, and IL-6, its interaction with IL-17 signaling, and its role in controlling osteoclast differentiation and alveolar bone resorption highlight its broader relevance in the pathogenesis of periodontitis. Despite growing interest in salivary and GCF levels of IL-10 and cortisol as non-invasive biomarkers, their clinical applicability remains to be validated. Variability in study design, inconsistencies in sampling methodology, and heterogeneity across patient cohorts have contributed to limited reproducibility and inconclusive diagnostic utility. In the future, it will be essential to implement standardized biomarker measurement protocols and define robust associations between these molecular indicators and disease severity, progression, and treatment response.

Future research should also focus on longitudinal studies that integrate stress-related biomarkers with genetic and immunological profiling to enhance our understanding of individual susceptibility. Therapeutic strategies that target the IL-10 pathway, whether through local delivery, modulation of regulatory B cells, or interventions aimed at restoring cytokine balance, hold promise for mitigating inflammation and preventing ABL. However, more fundamental animal and clinical studies on the interplay of IL-10 and cortisol in periodontitis patients experiencing chronic stress should be conducted to identify the key players in the complex interaction of the neuroendocrine and immunological response to stress in periodontitis patients and other inflammatory diseases. Recognizing the interplay between psychological stress, immune dysregulation, and tissue destruction offers a more comprehensive understanding of periodontitis and may open new avenues for prevention and personalized care.

## Open Access

This article has been published open access under our Subscribe to Open programme, made possible through the support of our subscribing institutions, learn more here: https://portlandpress.com/pages/open_access_options_and_prices#conditional

## Abbreviations

ACTH: adrenocorticotropic hormone
Breg: regulatory B cells
CAL: clinical attachment level
CRH: corticotropin-releasing hormone
CRH-R1: corticotropin-releasing hormone receptor-1
CRH-R2: corticotropin-releasing hormone receptor-2
CRH-R2: corticotropin-releasing hormone receptor-2
CgA: chromogranin A
CpG: cytosine-phospho-guanine
DHEAS: dehydroepiandrosterone sulfate
E: epinephrine
GCF: gingival crevicular fluid
GR-α: glucocorticoid receptor-α
GR-α: glucocorticoid receptor-α
HPA axis: hypothalamic–pituitary–adrenal axis
IFN-γ: interferon gamma
IL-6: interleukin-6
IL-10: interleukin-10
IL-17: interleukin 17
IL: 10R - interleukin-10 receptor
IL-10Rß: IL-10 receptor beta
IL-10Rα: IL-10 receptor alpha
IL-1β: interleukin-1 beta
NE: norepinephrine
NF-κB: nuclear factor kappa-light-chain-enhancer of activated B cells
OPG: osteoprotegerin
RANKL: receptor activator of nuclear factor kappa-Β ligand
ROS: reactive oxygen metabolites
SNPs: Several single-nucleotide polymorphisms
SNS: sympathetic nervous system
StAR: steroidogenic acute regulatory protein
TGF-β1: transforming growth factor-beta 1
TNF-α: tumor necrosis factor-alpha
3β-HSD: 3β-hydroxysteroid dehydrogenase
